# 967. Strength Recovery After Severe Burn Injury: Characterizing ICU-Acquired Weakness from Discharge to Follow-Up

**DOI:** 10.1093/jbcr/irag033.564

**Published:** 2026-04-06

**Authors:** Audrey M O'Neil, Leigh J Spera

**Affiliations:** Richard M Fairbanks Burn Center, Indianapolis, IN; Indiana University/Eskenazi Health, Indianapolis, IN

## Abstract

**Introduction:**

ICU-acquired weakness (ICUAW) affects up to 50% of long-stay ICU patients and is associated with prolonged disability, impaired quality of life, and increased healthcare utilization. ICUAW is typically assessed using the Medical Research Council (MRC) sum score, which evaluates strength in shoulder abduction, elbow flexion, wrist extension, hip flexion, knee extension, and ankle dorsiflexion. While the prevalence of ICUAW in general ICU populations is well documented, it remains unclear in burn survivors. Patients with large TBSA burns undergo significant muscle catabolism, highlighting the need for longitudinal musculoskeletal assessment, as loss of lean body mass contributes to acute morbidity and delays recovery. This study aimed to determine the prevalence of ICUAW in burn survivors at hospital discharge and to characterize its persistence post-discharge.

**Methods:**

Adult burn patients with >20% TBSA full-thickness burns admitted between January–August 2025 were included, representing a population with ICU-level care and resuscitation requirements. Strength was assessed within the week prior to discharge using the MRC sum score and grip strength testing when feasible. Outpatient follow-up assessments were completed during therapy sessions, documenting persistence of weakness relative to time since injury.

**Results:**

Twelve burn survivors were included (mean age 45 ± 17.2 years, 83% male, mean TBSA 43% ± 19.3, mean LOS 40 ± 23.8 days). At discharge, 58% (n = 7) met criteria for ICUAW (MRC sum score < 48/60). The mean MRC score was 44 ± 10.4, with grip strength testing feasible in 8 patients; 75% (n = 6) were below predicted norms. At follow-up (n = 10, median 110 days post-burn), only 1 patient (10%) continued to meet ICUAW criteria. Mean MRC scores improved to 54.4 ± 6.9, with 40% (n = 4) achieving full recovery (MRC ≥ 60). The most affected joints were wrist extension and hip flexion (mean 3.56 and 3.42). Barriers to testing included contractures, amputations, and pain, most often limiting assessment of the wrist, shoulder, and ankle. Trends suggested higher ICUAW prevalence with larger TBSA, older age, and mechanical ventilation.

**Conclusions:**

Burn survivors demonstrated a notable prevalence of ICU-acquired weakness at discharge, despite high therapy standards and early rehabilitation practices in verified burn centers. Many recovered quickly post-discharge, suggesting faster strength restoration compared to general ICU populations. However, strength testing in burn survivors is frequently limited by contractures and pain, warranting further study of the most feasible methods for this population.

**Applicability of Research to Practice:**

Routine screening for ICUAW in burn survivors should be integrated into discharge and follow-up care, with assessments tailored to the unique challenges of burn recovery to optimize functional outcomes.

**Funding for the study:**

N/A.

